# Sex-Linked Pheromone Receptor Genes of the European Corn Borer, *Ostrinia nubilalis,* Are in Tandem Arrays

**DOI:** 10.1371/journal.pone.0018843

**Published:** 2011-04-22

**Authors:** Yuji Yasukochi, Nami Miura, Ryo Nakano, Ken Sahara, Yukio Ishikawa

**Affiliations:** 1 Insect Genome Research Unit, National Institute of Agrobiological Sciences, Tsukuba, Ibaraki, Japan; 2 Department of Agricultural and Environmental Biology, Graduate School of Agricultural and Life Sciences, The University of Tokyo, Tokyo, Japan; 3 Laboratory of Applied Molecular Entomology, Graduate School of Agriculture, Hokkaido University, Kita-ku, Sapporo, Japan; University of Missouri, United States of America

## Abstract

**Background:**

Tuning of the olfactory system of male moths to conspecific female sex pheromones is crucial for correct species recognition; however, little is known about the genetic changes that drive speciation in this system. Moths of the genus *Ostrinia* are good models to elucidate this question, since significant differences in pheromone blends are observed within and among species. Odorant receptors (ORs) play a critical role in recognition of female sex pheromones; eight types of OR genes expressed in male antennae were previously reported in *Ostrinia* moths.

**Methodology/Principal Findings:**

We screened an *O. nubilalis* bacterial artificial chromosome (BAC) library by PCR, and constructed three contigs from isolated clones containing the reported OR genes. Fluorescence *in situ* hybridization (FISH) analysis using these clones as probes demonstrated that the largest contig, which contained eight OR genes, was located on the Z chromosome; two others harboring two and one OR genes were found on two autosomes. Sequence determination of BAC clones revealed the Z-linked OR genes were closely related and tandemly arrayed; moreover, four of them shared 181-bp direct repeats spanning exon 7 and intron 7.

**Conclusions/Significance:**

This is the first report of tandemly arrayed sex pheromone receptor genes in Lepidoptera. The localization of an OR gene cluster on the Z chromosome agrees with previous findings for a Z-linked locus responsible for *O. nubilalis* male behavioral response to sex pheromone. The 181-bp direct repeats might enhance gene duplications by unequal crossovers. An autosomal locus responsible for male response to sex pheromone in *Heliothis virescens* and *H. subflexa* was recently reported to contain at least four OR genes. Taken together, these findings support the hypothesis that generation of additional copies of OR genes can increase the potential for male moths to acquire altered specificity for pheromone components, and accordingly, facilitate differentiation of sex pheromones.

## Introduction

Evolution of genes responsible for sex pheromone communication in moths is an attractive model for investigating the relationship between the divergence of genes and mechanisms of speciation. Release of sex pheromones from female moths is believed to play a critical role in species recognition [1]–[2]. However, little is known about the changes that have occurred in the genomes of newly derived species that use pheromones different from the ancestral one.

Sex pheromones of moths are usually a blend of a few compounds synthesized from common fatty acids through desaturation, chain shortening and other modifications [3], and some mutations of genes involved in the pheromone biosynthesis pathway cause changes in pheromone composition [4]. An important question is how the pheromone recognition system in males can adapt rapidly to the changes that have occurred in the female pheromone biosynthetic pathway.

The genus *Ostrinia*, which includes the European corn borer, *Ostrinia nubilalis*, and the Asian corn borer, *Ostrinia furnacalis*, is an excellent model for studying the evolution of the pheromone biosynthesis and recognition systems, since various species show distinct differentiation in sex pheromones despite a relatively short period after speciation [5]. Sex pheromones of nine *Ostrinia* species have been characterized to date and six compounds have been found as the components of female sex pheromones (Fig. S1) [6]. *O. nubilalis* and its closely related congener, *Ostrinia scapulalis*, are unique in showing intraspecific variations in their pheromone blends; Z-type, I-type (hybrid), and E-type (Fig. S1) [7]–[9]. A major advantage of using the *Ostrinia* moths is that genetical analysis can be conducted by interspecific crosses and intraspecific crosses between different pheromone races. For example, crosses between E- and Z-strains of *O. nubilalis* revealed that the genetic factor responsible for the difference in female pheromone blend production is autosomal [10]–[11]. Recently, this autosomal locus was shown to be an allelic variation in a fatty-acyl reductase gene which is specifically expressed in the pheromone gland [4].

Similar autosomal loci controlling the pheromone blend were identified by crossing other combinations of *Ostrinia* species [12]–[13]. Although both *O. scapulalis* and *O. furnacalis* have genes encoding Δ11-desaturase and Δ14-desaturase, key enzymes in pheromone biosynthesis, only Δ11-desaturase is functionally expressed in *O. scapulalis*, [14] and so is Δ14-desaturase in *O. furnacalis* [15]. We have shown that transcription of mRNA from these desaturase genes occurs species specifically [16], which might be under the control of an autosomal locus responsible for the difference in pheromone blend.

By contrast, the locus responsible for male behavioral response to sex pheromone was reported to be Z-linked in *O. nubilalis* [10]–[11], [17]. The most likely candidates for this locus are pheromone receptors. A lepidopteran pheromone receptor was first identified as an odorant receptor (OR) specific to bombykol, a pheromone component of the silkworm, *Bombyx mori* [18]–[20]; ORs of four other moths showed responses to major pheromone components of their own [21]–[22]. It was reported recently that a locus responsible for the differential male response to pheromone compounds between *Heliothis subflexa* and *H. virescens* was linked to four OR genes [23].

We have subsequently reported isolation of genes encoding male-specific (OR1, 3–6, 8) and non-male-specific (OR7) ORs from eight *Ostrinia* moth species [24]–[25]. These OR genes show high similarity to known lepidopteran sex pheromone receptor subfamily genes [24]–[25]. When co-expressed with an Or83b homologue (OscaOR2), some ORs (OscaOR1, 3–5) of E-type *O. scapulalis* were observed to respond to several pheromone components used by *Ostrinia* moths; however, the specific response of males to their own pheromone blend could not be explained by the specificity of the observed ORs alone [24]–[25]. Wanner and colleagues independently reported isolation of five OR genes from Z-race *O. nubilalis,* four of which were consistent with those in our reports [26]; however, the gene names (hereafter, abbreviated as *OnOr1–5*) were not identical to ours (hereafter, abbreviated as *OnubOR1–8*) except for the OR2 gene. The one not found in *O. scapulalis*, *OnOr6*, was reported to be highly specific for (*Z*)-11-tetradecenyl acetate, the main component of the Z-race *O. nubilalis* pheromone blend [26].

Here, we report the chromosomal mapping and genomic organization of the OR genes described above. We screened an *O. nubilalis* BAC library for clones containing OR genes using cDNA sequences from *O. scapulalis*, which we subsequently used for FISH analysis in *O. nubilalis* and sequence determination. At least seven OR genes were in tandem arrays on the Z chromosome of *O. nubilalis*; a 181-bp direct repeat sequence was conserved among four of them. This is the first report of clustering of lepidopteran sex pheromone receptor subfamily genes. The chromosomal region where the cluster was located, determined by FISH analysis, was orthologous to *BmOr1*, a sex pheromone receptor gene of *B. mori*.

## Results

### Isolation of *O. nubilalis* BAC clones containing OR genes

To characterize the genomic organization of the *O. nubilalis* OR genes, we isolated BAC clones containing OR genes from an *O. nubilalis* BAC library by PCR-based screening in the same manner as described previously [27]. Since we had started the screening before determining cDNA sequences of the *O. nubilalis* OR genes, we used the *O. scapulalis* OR genes for designing primers. Consequently, we could isolate one or more positive clones from the library for each gene (Table S1).

The *OnubOR2 gene,* a single *O. nubilalis* ortholog of *OscaOR2,* was localized on one BAC clone, 07H10. Four BACs containing exons 7–8 of the previously reported *OnOr6* gene [26] were identified, two of which also contained exons 1–3 (Table S1). Additional OR genes were co-localized on two other groups of BACs using primers derived from *O. scapulalis* cDNAs, indicating that they were clustered. One group of three clones was found to contain both of the *OscaOR1*/*3* genes and six clones were isolated by primers for the *OscaOR4*–*8* genes (Table S1). With the exception of *OnOr6* which had already been published [26], we named each gene found on the *O. nubilalis* BAC clones according to the species from which the primer sequences were derived (Table S2).

### Chromosomal locations of OR genes revealed by BAC-FISH analysis

Although the *O. nubilalis* OR genes were localized to four BAC contigs, this did not necessarily mean that these clones were separately located. To identify chromosomal locations of the *OnubOR1*–*8* and *OnOr6* genes, we performed BAC-FISH analysis of the clones, 07H10, 14B20, 44E03 and 50D02, representing the *OnubOR2, OnubOR5/7, OnOr6* and *OnubOR1/3* genes, respectively (Table S2). We also selected 32P24 as a probe since the size of the introns of the *OnubOR7* gene on this clone was different from other clones.

**Figure 1:**
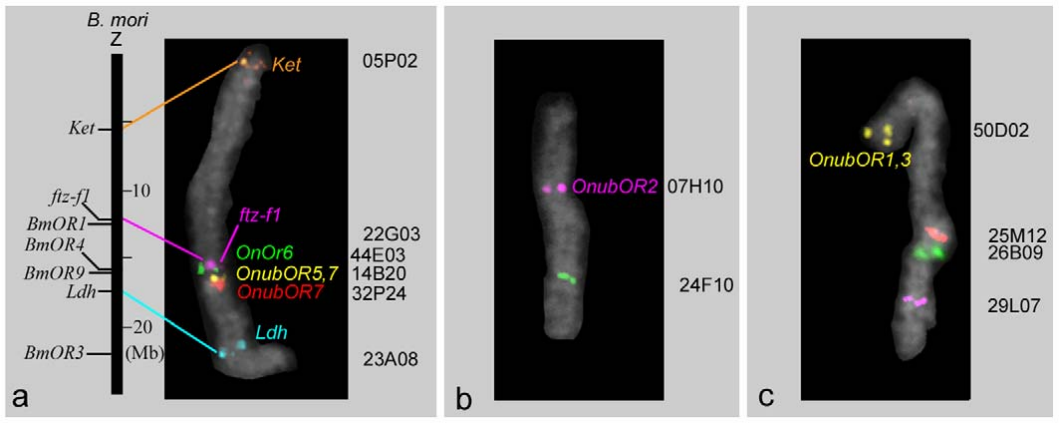
Identification of the chromosomal position of *O. nubilalis* OR genes by BAC-FISH analysis. BAC probe codes are shown on the right of each chromosome image (for details, see Table S2). Lines indicate correspondence of orthologous genes between *B. mori* and *O. nubilalis.* A) Comparison of the Z chromosomes. A vertical bar on the left side represents the *B. mori* Z chromosome drawn to relative scale taken from Kaikobase (http://sgp.dna.affrc.go.jp/KAIKObase/). B,C) Images of *O. nubilalis* chromosomes orthologous to *B. mori* chromosomes 16(B) and 23(C).

We previously reported the existence of significant synteny between *B. mori*, and the tobacco hornworm, *Manduca sexta,* by BAC-FISH [27]. In a parallel study, we isolated BACs containing *O. nubilalis* orthologs of *B. mori* genes [28] which could be used as specific probes for each chromosome. We first examined whether any OR genes were Z-linked since both the *Resp* locus responsible for male behavioral response to sex pheromone [11], [17] and the *BmOr1* gene encoding a major sex pheromone receptor in *B. mori* are located on the Z chromosome [18]. BACs containing Z-linked *O. nubilalis* genes encoding kettin (*Ket*), FTZ-F1 (*ftz-f1*) and lactate dehydrogenase (*Ldh*) were used as markers specific for the Z chromosome (Table S2). Signals of the 14B20, 32P24 and 44E03 probes were detected from neighboring positions on approximately one third of the Z chromosome, distal to the *ftz-f1* gene, indicating that the *OnubOR4*–*8* and *OnOr6* genes comprised a large gene cluster (Fig. 1A). The locations of 14B20 and 32P24 were not identical (Fig. 1A), suggesting that these clones contained different copies of the *OnubOR7* genes.

Two other clones containing the *OnubOR2* and *OnubOR1/3* genes were found on autosomes. Since sequences of *Or83b* co-receptor genes are well conserved among lepidopterans [22], we speculated that the chromosomal locations of *OnubOR2* and its *B. mori* ortholog, *BmOr2,* were also conserved. Since the *BmOr2* gene was mapped onto chromosome 16 of *B. mori*, we used 24F10 harboring two genes (EL929838 and EL929540) whose *B. mori* orthologs are located on this linkage group as markers (Table S2) together with 07H10 containing the *OnubOR2* gene in a FISH analysis. As expected, 07H10 and 24F10 were co-localized on the same chromosome (Fig. 1B).

For mapping of the *OnubOR1* and *3* genes, genetic analysis was necessary since their *B. mori* orthologs had not been identified by sequence comparison [25]–[26]. Recombination by crossing-over does not occur in lepidopteran oogenesis so that genes on the same chromosome always co-segregate. This makes it possible to test whether markers belong to the same linkage groups by using backcross progeny from a mating of an F1 female and a homozygous parental male (termed “BF1”). Using twenty-four BF1 progeny between *O. nubilalis* and *O. scapulalis*, we found that the *OnubOR1* and *3* genes co-segregated with a gene (Accession no. FS438672) whose *B. mori* orthologs are located on *B. mori* chromosome 23 (Table S2). Therefore, three BACs physically mapped onto the *O. nubilalis* counterpart of *B. mori* chromosome 23 were used as probes for FISH analysis with 50D02 representing the *OnubOR1* and *3* genes (Table S2). The signal from 50D02 was detected near the end of the chromosome where the signals from three positive controls were located (Fig. 1C).

### Duplication of the *OR5* group genes

To characterize the genomic organization of the OR genes, we sequenced the BAC clones (08K04 and 11K16) that contained *O. nubilalis* OR genes other than *OnubOR2* and *OnOR6*.

In the previous report, we found multiple *OR5* and *OR6* genes for each *Ostrinia* species; however, *OR5* and *OR6* genes form a single clade without separating into *OR5* and *OR6* groups [25]. Thus, we classified these genes as variants of a newly defined *OR5* group, which was also useful for avoiding confusion with the *OnOr6* gene independently reported by Wanner et al. [26]. We also identified the *O. scapulalis* orthologue of the *OnOr6* gene and confirmed that it was transcribed in male antennae.

**Figure 2:**
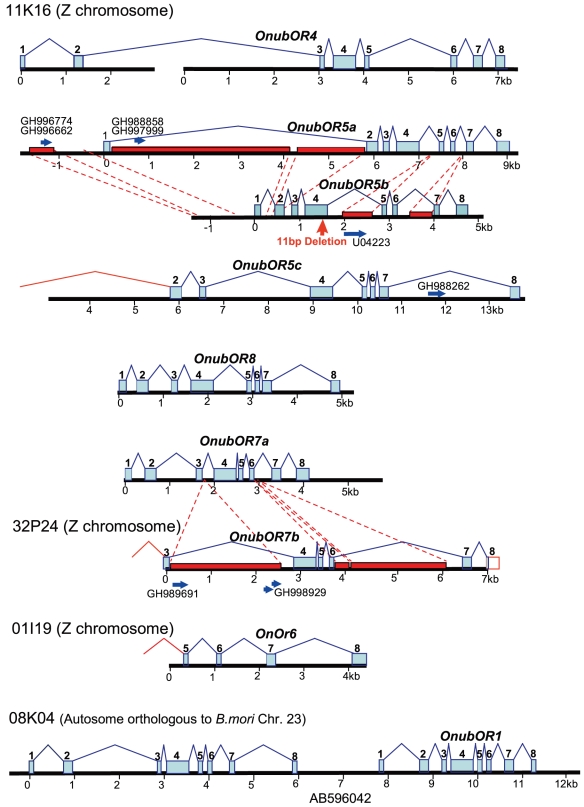
Structure of *O. nubilalis* OR genes determined in this study. Numbers under bars indicate distance from putative transcriptional start sites (kbp) when all the exons and introns are identified. Blue squares represent exons and dotted squares indicate unidentified exons. Dotted lines show the correspondence of conserved sequences between the *OnubOR5a/b, 7a/b* genes and red squares indicate sequences solely appearing in one of the genes. Blue arrows represent putative repetitive elements found in *O. nubilalis* ESTs and genomic sequences.

Sequencing of the BAC clone 11K16 revealed three apparently independent copies of the *OnubOR5* gene. Exons for two of them were nearly identical to the *OscaOR5*, *OnubOR6* [25] and *OnOr1* [26] genes, so we designated them as *OnubOR5a* and *5b.* The *OnubOR5b* gene contained an 11-bp deletion in exon 4 which caused a frameshift mutation leading to a truncated product (Fig. 2).

Significant similarity was observed between the *OnubOR5a* and 5*b* genes including introns and flanking regions (Fig. 2). Non-conserved sequences were inserted into the upstream region and intron 1 of the *OnubOR5a* genes and introns 4 and 6 of the *OnubOR5b* genes (Fig. 2). Some of the additional sequences showed high partial similarity to *O. nubilalis* ESTs and genomic sequences, suggesting insertion of repetitive elements (Fig. 2). For example, the insertion into intron 4 of the *OnubOR5b* gene showed high similarity to intron 2 of the alpha-amylase gene (Accession no. U04223) and to an EST of the spruce budworm, *Choristoneura fumiferana* (Accession no. FC952039). Since the superfamily Tortricoidea to which *C. fumiferana* belongs is estimated to have diverged from advanced Lepidoptera soon after the lepidopteran radiation [29]–[30], the evolutionary origin of the insertion sequence is old. However, no similar sequences were found in the *B. mori* genome database nor in published sequences of other lepidopteran species.

**Figure 3:**
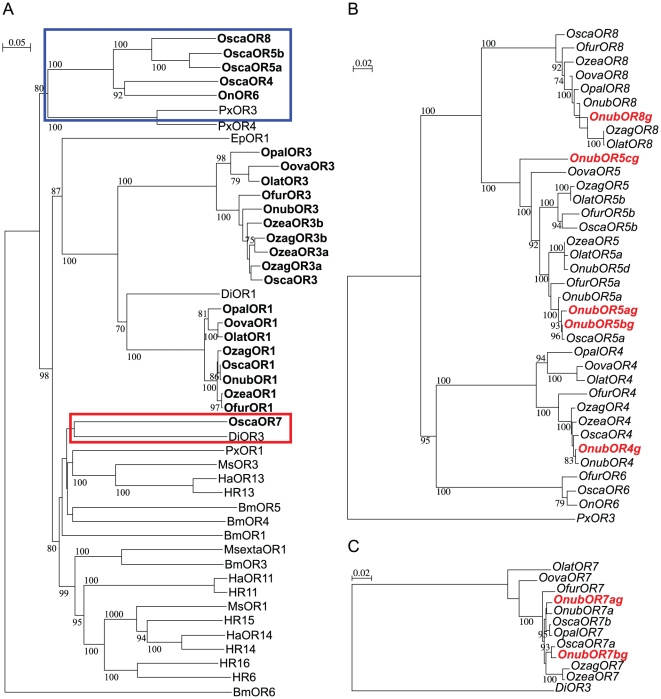
Phylogenetic relationship of lepidopteran OR genes belonging to the sex pheromone receptor subfamily. Blue and red boxes in panel A indicate regions separately shown in panels B and C, detailed phylogenetic trees of *OR4, 5, 8* and *OnOr6* genes (B) and OR7 genes (C) of *Ostrinia* moths. Detailed information of genes used for comparison is listed in Table S4.

The third copy, designated as *OnubOR5c,* showed a relatively low degree of similarity to the known *Ostrinia OR5* genes; nevertheless, it was included in the *OR5* clade (Fig. 3). Neither deletions nor nonsense mutations were found in it. In addition, we could not identify exon 1 of the gene, although more than 5 kb of genomic sequences was determined for the putative region corresponding to it (Fig. 2).

### Tandem repeats spanning exon 7 and intron 7 of the *OnubOR5/8* genes

**Figure 4:**
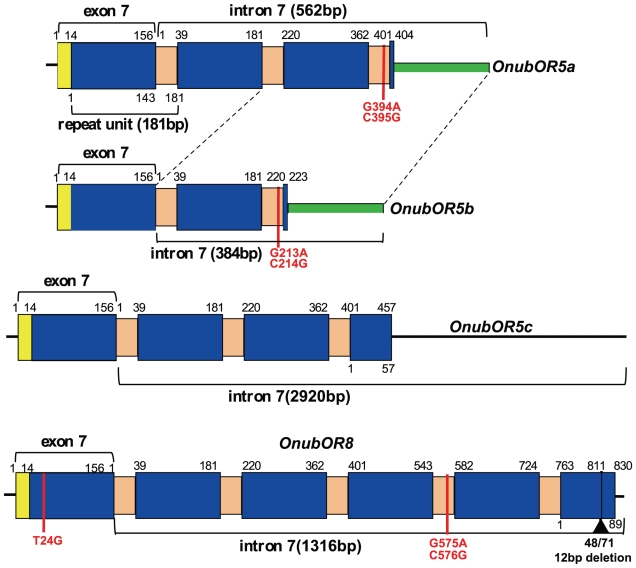
The structure of the 181-bp repeat sequences. Yellow squares indicate exon 7 sequences not included in the repeat. Blue squares indicate sequences shared with exon7 and repeats in intron 7. Pink squares indicate intron7-specific sequences. Green squares indicate sequences conserved among the *OnubOR5a,b* genes. Red vertical lines show base substitutions. Black vertical line indicates a putative deletion in the last truncated repeat of the *OnubOR8* gene.

We found 181-bp tandem repeats located from exon 7 to the 5′- end of intron 7 of the *OnubOR5a,b,c* genes. The repeat unit was composed of a 143-bp portion identical to nucleotides 14–156 of exon 7 and a 38-bp portion unique to the repeat. The *OnubOR5a,b,c* genes contained three, two and three complete repeats followed by a truncated exon 7-like sequence (Fig. 4).

The *OR4,5*,*8* genes of several *Ostrinia* moths and the *OnOr6* gene formed a definite clade which did not include any known OR genes of other Lepidoptera (Fig. 3A) [25]–[26]. This raised the possibility that the *OnubOR4,8* and *OnOr6* genes were also duplicated or contained the 181-bp repeat, so we determined their genomic sequences. Consequently, we found that the *OnubOR8* gene contained five complete 181-bp repeats and one that was truncated (Fig. 4). No repeats were found in the *OnubOR4* gene, nor did we find additional copies of the *OnubOR4,8* genes within clone 11K16 (Fig. 2). We also determined the genomic sequence containing exons 5–8 of the *OnOr6* gene by PCR amplification of BAC clone 01I19 (Fig. 2); however, the 181-bp repeat was not present. We also confirmed that the *O. scapulalis* orthologue of the *OnOr6* gene was transcribed in male antennae, and designated it as *OscaOR6* (Fig. 3B).

It is noteworthy that there was one base substitution in exon 7 of the *OnubOR8* gene despite the identical repeat sequence and a 2-bp base substitution was conserved among all of the *OnubOR5a,b* and *8* genes (Fig. 4). No significant similarity was observed between exon 8 of the *OnubOR8* and *OnubOR5a,b,c* genes, in contrast to extensive similarities through exons 1–7.

### Genome sequencing encompassing exons 3 and 4 of the *OR5b* gene

To confirm whether deletions found in the BAC sequence of 11K16 occurred in other individuals, we determined genomic sequences encompassing exons 3–4 of the *OnubOR5b* genes using other BAC clones, as well as in one female and four male BF1 progeny from the crosses between *O. nubilalis* and *O. scapulalis* used for linkage analysis. The sequence of 53I05 was identical to 11K16. Altogether, we found one allele from clone 14B20 and six alleles from BF1 progeny, which were not identical to 11K16 (Fig. S2). The 11-bp deletion was not observed in other alleles; however, four of them harbored one of three indels in exon 4 causing frameshift mutations (Fig. S2).

### Genome organization of the *OnubOR7* genes

As described above, two copies of the *OnubOR7* genes were found separately on clones 11K16 and 32P24. The sequence of the gene on 11K16 was determined in the same manner as described above, and eight exons corresponding to the cDNA sequence of the *OscaOR7* gene were found, which we designated as *OnubOR7a* (Fig. 2).

Direct amplification was carried out on the gene on 32P24, designated as *OnubOR7b,* using primers designed from exon sequences of the *OscaOR7* gene. Six exons (putative exons 3–8) and five introns (putative introns 3–7) were determined. Sequence identity of the *OnubOR7b* gene to the *OscaOR7* gene was clearly higher than that of the *OnubOR7a* gene (Fig. 3C). We had previously identified a transcript nearly identical to the *OnubOR7a* gene from male antennae of *O. scapulalis* (Fig. 3C); however, we had interpreted it as an intraspecific variation of a single locus. We renamed the previously reported one as *OscaOR7a* and designated the other as *OscaOR7b* (Table S3), since it was likely that two types of *OR7* genes were expressed in *O. scapulalis.*

The genomic sequences of the *OnubOR7a* and *b* genes were highly conserved including most of the introns; however, two observed differences in intron size were due to insertions detected in introns 3 and 6 of the *OnubOR7b* gene (Fig. 2). Interestingly, portions of both ends of intron 3 were very similar to *O. nubilalis* ESTs (Accession nos. GH989691 and GH998929) (Fig. 2). Direct repeats on the 3′- end also showed significant similarity to numerous genomic sequences in *B. mori* and to ESTs of a butterfly, *Bicyclus anynana,* suggesting that this insertion was a transcribed repeated sequence with conserved motifs (Fig. 2).

### Genome sequence of the *OnubOR1* and *OnubOR3* genes

Since the *OscaOR1* and *OscaOR3* genes show significant sequence similarity [25] and their *O. nubilalis* orthologs were co-localized on the same BAC clones (Table S1), we speculated that they were also created through a gene duplication and remained closely located. Thus, we carried out extensive sequencing to construct a single contig. Ultimately, we determined approximately 13.8-kb genomic sequence and revealed that the *OnubOR3* gene was located in a 1.85-kb interval upstream of the *OnubOR1* genes (Fig. 2).

### Tandem array of the OR genes on the Z chromosome

**Figure 5:**
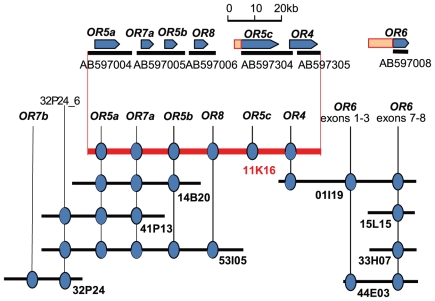
The order of *O. nubilalis* OR genes on the Z chromosome. Pentagons represent the position, length and transcriptional orientation of OR genes. Bars under the pentagons indicate the sequenced contigs of clones 11K16 and 01I19.

Finding a tandem array of the *OnubOR1* and *3* genes strengthened our speculation that the OR genes on 11K16 might also be in a tandem array. To identify the complete organization of the OR gene cluster, we continued to sequence the intergenic regions between the OR genes described above. Consequently, we determined two scaffolds of more than 42.2-kb and 27.9-kb for clone 11K16, the former (Accession nos. AB597004–AB597006) containing the *OnubOR5a-7a-5b-8* genes and the latter (Accession nos. AB597304–AB597305) containing the *OnubOR5c-4* genes in tandem arrays (Fig. 5). Since the 5′-end of AB597004 and the 3′-end of AB597305 were consistent with the distal ends of clone 11K16, the order of the six OR genes was *OnubOR5a-7a-5b-8-5c-4* (Fig. 5). We carried out PCR amplification against clones 01I19, 11K16 and 44E03 to confirm whether they overlapped, and found that clone 01I19 contained the *OnubOR4* gene (Fig. 5). Judging from the overlaps with other BAC clones, the overall gene order was *OnubOR5a-7a-5b-8-5c-4-OnOr6* (Fig. 5).

A genomic marker, 32P24_6, designed from the genomic sequence of a randomly selected subclone of 32P24 was also positive for 41P13 and 53I05, indicating that the *OnubOR7b* gene was located upstream of the OR gene cluster (Fig. 5). This result was consistent with the FISH results indicating that the *OnubOR7b and OnOr6* genes were on opposite ends of the OR gene cluster (Fig. 1).

## Discussion

The number and chromosomal distribution of insect OR genes vary among species [31]–[32]. The first sequenced insect, *Drosophila melanogaster,* has 62 OR genes which are scattered throughout the genome [33]. In contrast, other sequenced Diptera, the mosquitoes, *Anopheles gambiae* and *Aedes aegypti,* have more OR genes, 79 and 131, respectively, which are in many cases clustered [34]–[35]. There are also significantly many clustered OR genes, 163 and 299, in the honeybee, *Apis mellifera*, and the red flour beetle, *Tribolium castaneum*, representing Hymenoptera and Coleoptera [36]–[37].

*B. mori* was recently reported to have 66 OR genes [38], many of which are dispersed throughout the genome (Table S4). Most clusters contain pairs and triplets, but neither the *BmOr1* nor the *BmOr3* genes encoding pheromone receptors are clustered (Table S4). We found at least eight genes in the cluster on the Z chromosome described here (Fig. 5), which is the largest lepidopteran OR gene cluster reported to date. By linkage analysis using two congeneric moths, *H. subflexa* and *H. virescens,* Gould and colleagues revealed that the locus responsible for male response to female sex pheromone contains at least four OR genes (HR6,14–16 in Fig. 3A) [23]. These ORs are more closely related to each other than to any other *Heliothis* ORs [23], suggesting that the tightly linked genes were generated by gene duplication. These observations reinforce the attractive hypothesis that significant differences in sex pheromone blends of the *Ostrinia* and *Heliothis* moths are associated with the clustered OR genes.

It is likely that mutations in genes of the female pheromone biosynthesis pathway causing changes in pheromone blend must precede those of the male response system [39]. For example, Lassance and colleagues showed that intraspecific variations in the sex pheromone blend of *O. nubilalis* could be explained by the sequence divergence of a fatty-acyl reductase gene that affected its substrate specificity [4]. Pre- or immediate adaptation of male moths to such sudden changes is critical for emergence of a population using novel pheromone blends, and it is unlikely that a great change in the specificity of pheromone receptors is caused by mutations at the same time as those in the pheromone biosynthesis pathway.

On the other hand, there are “rare” *Ostrinia* males (3–5%) that possess the ability to respond to a pheromone blend which is not effective for most individuals in the population [15], [40]–[41]; moreover, significant differences in olfactory neuron responsiveness have been observed between rare and normal males [42]–[43]. Tandemly arrayed genes are thought to be subject to birth-and-death evolution, in which new genes are created by duplication. Some of these are retained in the genome as functional genes, but others are inactivated or eliminated from the genome [44]. Adaptation to a novel pheromone blend might be gained by altered specificity of newly created genes which are not initially under high selective pressure.

Genome sequencing revealed the presence of novel OR genes, the *OnubOR5b* and *5c* genes, which had not been detected by RT-PCR analysis. As described above, the *OnubOR5b* gene seemed to be nonfunctional because insertions or deletions causing frameshift and truncation of the product occurred independently in several alleles (Fig. S2). Deletions similar to the *OnubOR5b* gene were observed in the strain used in the previous report [25]. Ninety-bp and 5-bp deletions were located in exon 4 of the *OnubOR5* (renamed *OnubOR5d*) and *OnubOR8* genes, respectively

The incongruence of the phylogenetic trees between the *OR5/8* genes and the mitochondrial gene cytochrome oxidase II (COII) (Fig. S1) or other OR genes (Fig. 3A) also supports the hypothesis that the *OR5/8* genes are undergoing a process of birth-and-death evolution. It is likely that the major *OR5/8* genes expressed in each species are not necessarily orthologous due to gene duplication and independent gene inactivation. Since detailed sequencing was performed only for the clone 11K16 in this study, other OR genes might be located outside of it. In fact, we could not find an *O. nubilalis* ortholog of the *OscaOR5b* gene (Fig. 3B) in any of the BAC clones we investigated.

It is possible that gene duplications occurred via the 181-bp repeat which was conserved among the *OR5/8* genes (Fig. 4), since the *OR4* gene lacking the repeat seems to be relatively stable and all the *Ostrinia* species examined retained functional *OR4* genes during speciation [25]. A number of inactivated copies of Δ11-desaturase genes were reported in *O. nubilalis* and *O. furnacalis,* and unequal crossover via a retrotransposon, *ezi*, was proposed to cause gene duplication [45]. Thus, we came up with a similar hypothesis that the 181-bp repeat enhances duplication of OR genes containing it. The presence of a 2-bp substitution which is conserved among the tandemly linked *OnubOR5a,b* and *8* genes is strong evidence for unequal crossovers via the 181-bp repeat, since it is very rare that such substitutions occurr independently. Recently, Heckel described the potential involvement of tandem gene duplication for the immediate creation of a novel phenotype in the sexual communication system [46]. The results reported here may be the first evidence supporting this idea. Further analysis of OR gene clusters including those of *Ostrinia* moths will reveal more details of the birth-and-death process of the *OR5/8* genes including ones which have been inactivated.

The *OR7* genes were also duplicated and both genes were transcribed in *O. scapulalis* (Fig. 3C). However, the mechanism of duplication seems to be different from that of the *OR5/8* genes, since repeats like the *OR5/8* genes were not present (Fig. 2). In the previous report, Osca7a showed no significant response to any of the pheromone compounds used by the *Ostrinia* moths, even though the *OR7* genes were more similar to the sex pheromone receptor genes of other Lepidoptera than the male-specific *Ostrinia* OR genes [25]. The *DiOR3* gene of the cotton caterpillar moth, *Diaphania indica*, similar to the *OR7* genes (Fig. 3A), was also expressed in antennae of both males and females [22]. Since *D. indica* belongs to Pyraloidea, the same superfamily as the genus *Ostrinia,* the *DiOR3* and *Ostrinia OR7* genes might have lost a male-specific expression pattern in the lineage leading to Pyraloidea.

The OR gene cluster revealed by FISH analysis was located at approximately one third of the distance from the end of the Z chromosome near the *ftz-f1* gene (Fig. 1A) which was consistent with the position of the *BmOr1* gene (Table S1, S3). Thus, it is possible that the *OR7* genes in the *Ostrinia* OR gene cluster and the *BmOr1* gene evolved from a common ancestral gene, although evidence of other orthologous genes located in the neighboring chromosomal region will be needed to verify this hypothesis. On the other hand, no OR genes similar to *OR4,5,8* or *OnOr6* have been isolated from other advanced Lepidoptera including *B. mori*, which has been fully sequenced [47]. *PxOR3* and *PxOR4* isolated from the diamondback moth, *Plutella xylostella*, are the only reported OR genes which formed a clade with the *OR4,5,8* and *OnOr6* genes (Fig. 3A, B). Since Yponomeutoidea, which includes *P. xylostella*, is thought to have diverged earlier than the split of Pyraloidea from other advanced Lepidoptera [29]–[30], it is likely that this type of OR gene was lost or inactivated in the lineage leading to *B. mori*.

The *OnubOR1* and *3* genes are autosomal (Fig. 1) and thus do not directly determine male pheromone behavioral response in *O. nubilalis* since the locus responsible for the response is reported to be Z-linked [10]–[11], [17]. This is consistent with our previous results indicating that OscaOR1 and OlatOR1 specifically responded to (*E*)-11-tetradecenol, a single pheromone component of *O. latipennis* [24], and OscaOR3 responded to a wide variety of pheromone components used in the *Ostrinia* moths [25]. Similarly, the locus responsible for electrophysiological response in *O. nubilalis* was previously thought to be autosomal [10]; however, a recent genetic study showed that the response of pheromone sensitive sensilla may be affected by both autosomal and Z-linked genes [48]. Therefore, the *OnubOR1* and *3* genes might also be involved in pheromone recognition.

The *Ostrinia OR1* genes showed higher similarity to the *DiOR1* gene which encodes a putative sex pheromone receptor of *D. indica* [22], compared with the *Ostrinia OR3* genes. This raises a question whether the locus responsible for male pheromone response is autosomal or sex-linked in *D. indica* or *O. latipennis*. As seen with OscaOR1 and OlatOR1, the existence of receptors specifically responding to a pheromone compound is necessary but not sufficient to explain species specificity in the pheromone communication system.

A remaining question is whether the OR gene cluster reported here is equivalent to the *Resp* locus responsible for the *Ostrinia* male behavioral response to sex pheromone. In molecular genetic mapping experiments, Dopman and colleagues reported that the gene order is *Ket-Tpi* (triosephosphate isomerase)*-Ldh-Resp*, with *Ket* at one end and *Ldh* in the middle of the map [17]. However, our FISH results indicated that *Ldh* was near the end of the Z chromosome far removed from *Ket* (Fig. 1). The fact that polymorphisms in *Tpi* are consistent with the behavior of E- and Z-type pheromones [17] also suggests that markers were incorrectly ordered in the genetic map and *Resp* is actually located near *Tpi* which resides between *Ket* and *Ldh.* These results are not in conflict with our assumption that the OR gene cluster plays a critical role in determining male behavioral response to sex pheromone. Recent findings that male electrophysiological response is affected by Z-linked genes [48] strengthens the possibility that the *Resp* locus is equivalent to the OR gene cluster.

Detailed genetic dissection is needed to reveal the relationship between the OR gene cluster and the *Resp* locus; however, the available data are insufficient to identify the gene responsible for the *Ostrinia* male behavioral response. The existence of tightly linked multiple male-specific pheromone receptor genes will make it difficult to clarify which factor is definitive in determining male behavioral response to sex pheromone, as reported in the *Heliothis* moths [23]. In addition, it is still unclear how signals mediated by multiple ORs with narrow or broad specificity to pheromone compounds [25]–[26] are finally recognized as the definitive stimulus. Integrated approaches combining genetics, genomics, evolutionary biology and neurobiology are needed to reveal the detailed mechanisms underlying this complex trait.

## Materials and Methods

### PCR-based screening of the BAC library

An *O. nubilalis* BAC library, ON_Ba (average insert size 125kb, 36,864 clones), was obtained from the Clemson University Genomics Institute (Clemson, SC, USA). PCR-based screening of the library is described elsewhere [28]. The first screening was performed against DNA pools derived from 96 plates, using a mixture of 384 BAC-DNAs for each plate, followed by a second screening against DNA pools for 24 columns and 16 rows, each composed of mixtures of BAC-DNAs located in the same column or row. Primers used for the study were designed from cDNA sequences of *OscaOR1-8* of *O. scapulalis* and the *OnOR6* of *O. nubilalis* genes [24]–[26] (Table S1). As an exception, a single set of primer pairs was designed for the *OscaOR5* and *6* genes, since sequence similarity between them was too high.

### Crossing experiments

F1 females from matings between Z-type *O. scapulalis* females (originally collected in Matsudo, Japan) and Z-race *O. nubilalis* males (originally collected in Darmstadt, Germany) were backcrossed with Z-type *O. scapulalis* males, and genomic DNA was extracted from individual larvae and adults of the resultant BF1 progeny using an AquaPure Genomic DNA kit (Bio-Rad, Hercules, CA). DNA samples of twelve larvae and twelve adults (6 males and 6 females) were used for genotyping with the same PCR primers used for BAC isolation [28] in the same manner as described previously [49].

### BAC-FISH analysis

We followed the procedure described previously [27] for multi-color BAC-FISH. Chromosome spreads were prepared from pachytene spermatocytes of *O. nubilalis* larvae. BAC-DNA was extracted with a Plasmid Midi kit (QIAGEN, Hilden, Germany) and labeled with a fluorochrome using a Nick Translation System (Invitrogen Carlsbad, CA, USA). For each probe one of four fluorochromes [Green-dUTP, Orange-dUTP, Red-dUTP (Abbott Molecular Inc., Des Plaines, IL, USA), and Cy5-dUTP (GE Healthcare UK, Buckinghamshire, UK)], was used.

### Sequence determination of BAC clones

A shotgun library was constructed from equal amounts of BAC-DNAs from clones 08K04 and 11K16. Sonicated BAC-DNA ranging around 2 kb was inserted into the *Hin*c II site of plasmid pUC119. Shotgun clones were aliquoted into six 384-well microplates and DNA pools were made for 24 columns and 16 rows representing clones located in the same column or row. PCR screening was carried out against column and row DNA pools directly as described above.

Shotgun clones were first screened with primers used for BAC isolation. DNA templates were prepared using a DNA isolation kit (Kurabo, Japan), and sequenced with an ABI-3730*xl* DNA analyzer. Genome sequences assembled from overlapped shotgun clones isolated with the same primers were then used to design new primers to isolate clones located in the neighboring region. Remaining gaps were filled by direct PCR amplification of BAC-DNAs. PCR amplification was also performed against BAC clones, 32P24 and 01I19, to amplify flanking regions of exons of the *OnubOR7b* and *OnOr6* genes. The resultant PCR products were cloned using the pGEM-T easy Vector System (Promega, Madison, MA, USA) and used as sequencing templates.

In all, 229 end- or internal sequences of 143 shotgun clones and six PCR products were used for assembly of the 42.2-kb scaffold containing the *OnubOR5a-7a-5b-8* genes. Similarly, 168 sequences of 102 shotgun clones and three PCR products were used to assemble the 27.9-kb scaffold containing the *OnubOR5c-4* genes. The 13.8-kb genomic sequence containing the *OnubOR3-1* genes was constructed from 64 end-sequences of 41 shotgun clones. All sequences were submitted to DDBJ/GenBank/EBI Data Bank with accession numbers AB597004–AB597008, and AB597304–AB597305.

### Phylogenetic analysis

Deduced amino acid sequences (Fig. 3A) or nucleotide sequences (Fig. 3B, C) were used for comparison. The nucleotide and amino acid sequences were aligned using Clustal X [50]. The phylogenetic tree was constructed with the neighbor-joining method using the software PHYLIP 3.66 [51]. Branch support was assessed by the bootstrap test with 1000 re-samplings.

### Sequence determination of exons 3 and 4 of the *OR5b* gene

Amplification of genomic fragments containing exons 3 and 4 of the *OnubOR5b* gene was performed using BACs, 14B20, 41P13, 53I05 or genomic DNAs of BF1 progeny described above with PCR primers 5′-CGTTCACAGGTCCGTAGTA and 5′-ACTTTATCTCAGGCGACAT. Amplified fragments were then cloned using the pGEM-T easy Vector System (Promega) and used as templates for sequencing. All of the PCR products amplified from 41P13 corresponded to a portion of the *OnubOR5a* gene.

## Supporting Information

Figure S1 Phylogenetic relationships (left) and sex pheromone blends (right) of *Ostrinia* species. The phylogenetic tree was constructed based on mitochondrial COII gene sequences. The numbers near branches indicate bootstrap values. The size of circles represents a rough blend ratio. Z-type, I-type (hybrid), and E-type females of *O. scapulalis* and *O. nubilalis* produce mixtures of 3:97, 64:36, and 99:1 (E)- and (Z)-11-tetradecenyl acetates, respectively
(PDF)

Figure S2 Alignment of allelic genomic sequences encompassing exons 3 and 4 of the *OnubOR5b* gene (Accession Nos. AB596043–AB596049). Gray-shaded boxes indicate exons 3 and 4. Yellow-shaded letters indicate insertions or deletions causing frameshift mutations. Asterisks indicate nucleotides conserved among all the alleles.
(DOC)

Table S1 *O. nubilalis* BACs isolated by PCR-based screening.
(DOC)

Table S2 BAC probes used for FISH analysis.
(DOC)

Table S3 Detailed information of genes appearing in Figure 3 and summary of renaming of OR genes previously reported in ref. 25. “g” was added to names of genes deduced from genomic sequences.
(XLS)

Table S4 Chromosomal distribution of OR genes in *B. mori*.
(DOC)
