# Supplementary figures and images for: Sex-Linked Pheromone Receptor Genes of the European Corn Borer, Ostrinia nubilalis, Are in Tandem Arrays
Source: PLoS One. 2011 Apr 22;6(4):e18843. doi: 10.1371/journal.pone.0018843 (PMC3081303; doi:10.1371/journal.pone.0018843)

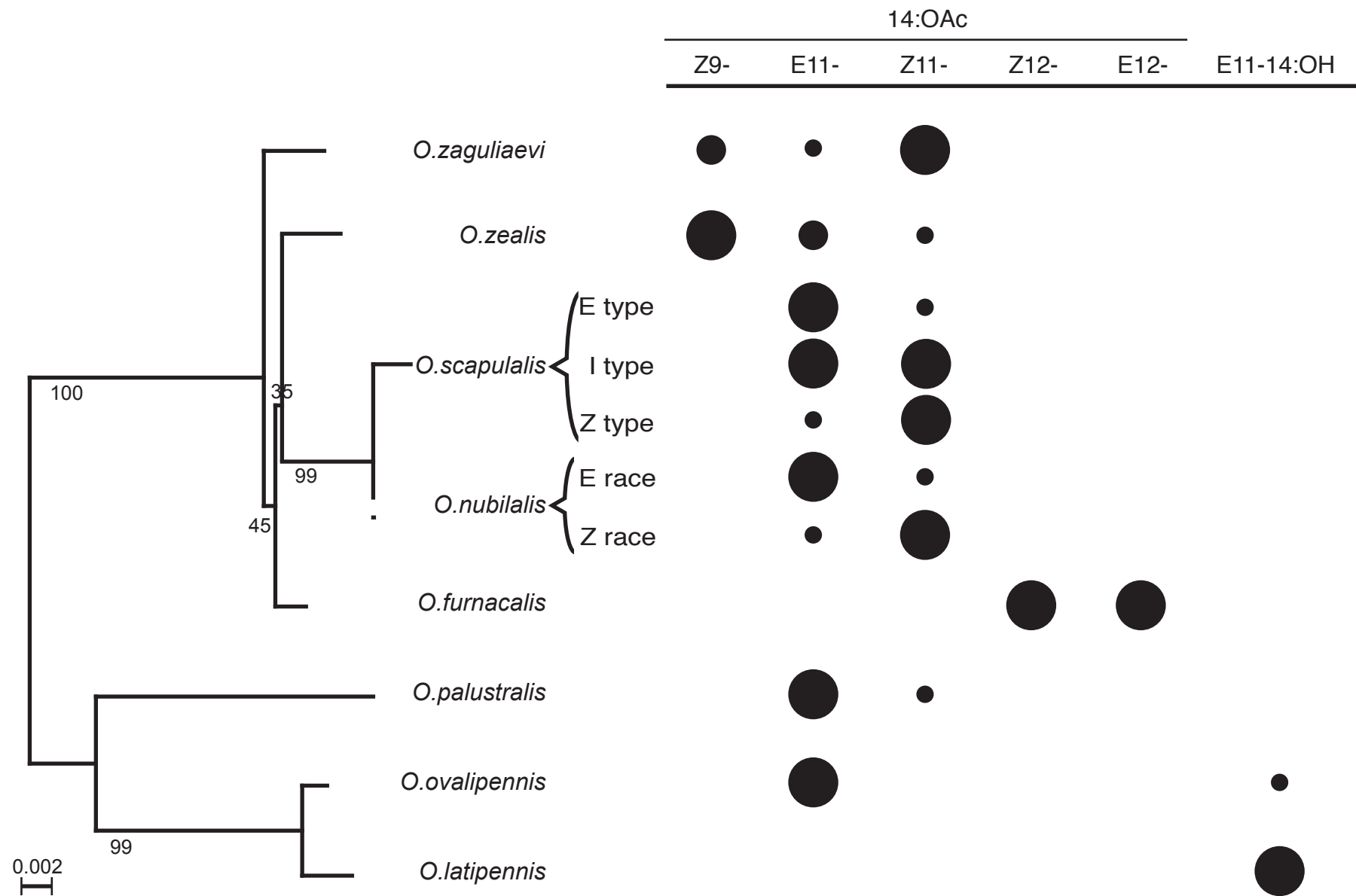

Supplement: Figure S1 — Phylogenetic relationships (left) and sex pheromone blends (right) of Ostrinia species. The phylogenetic tree was constructed based on mitochondrial COII gene sequences. The numbers near branches indicate bootstrap values. The size of circles represents a rough blend ratio. Z-type, I-type (hybrid), and E-type females of O. scapulalis and O. nubilalis produce mixtures of 3:97, 64:36, and 99:1 (E)- and (Z)-11-tetradecenyl acetates, respectively (PDF) [file pone.0018843.s001.pdf]
