# Supplementary material for: Sex-Linked Pheromone Receptor Genes of the European Corn Borer, Ostrinia nubilalis, Are in Tandem Arrays
Source: PLoS One. 2011 Apr 22;6(4):e18843. doi: 10.1371/journal.pone.0018843 (PMC3081303; doi:10.1371/journal.pone.0018843)
Supplement: Figure S2 — Alignment of allelic genomic sequences encompassing exons 3 and 4 of the OnubOR5b gene (Accession Nos. AB596043–AB596049). Gray-shaded boxes indicate exons 3 and 4. Yellow-shaded letters indicate insertions or deletions causing frameshift mutations. Asterisks indicate nucleotides conserved among all the alleles. (DOC) [file pone.0018843.s002.doc]

11K16 1 CGTTCACAGGTCCGTAGTATACTACCACAATTGCAGAACTATCATAAGTTGCTGCTAAAA 60

14B20 1 CGTTCACAGGTCCGTAGTATACTACCACAATTGCAGAACTATCATAAGTTGCTGCTAAAA 60

allele 74 1 CGTTCACAGGTCCGTAGTATACTACCACAATTGCAGAACTATCATAAGTTGCTGCTAAAA 60

allele 58 1 CGTTCACAGGTCCGTAGTATACTACCACAATTGCAGAACTATCATAAGTTGCTGCTAAAA 60

allele 64 1 CGTTCACAGGTCCGTAGTATACTACCACAATTGCAGAACTATCATAAGTTGCTGCTAAAA 60

allele 48 1 CGTTCACAGGTCCGTAGTATACTACCACAATTGCAGAACTATCATAAGTTGCTGCTAAAA 60

allele 53 1 CGTTCACAGGTCCGTAGTATACTACCACAATTGCAGAACTATCATAAGTTGCTGCTAAAA 60

************************************************************

11K16 61 TTTATAAATGTGATGCATTTGATGCATTCCACAAATAAAGGACCGTATTACAATCAGGTG 120

14B20 61 TTTATAAATGTGATGCATTTGATGCATTCCACAAATAAAGGACCGTATTACAATCAGGTG 120

allele 74 61 TTTATAAATGTGATGCATTTGATGCATTCCACAAATAAAGGACCGTATTACAATCAGGTG 120

allele 58 61 TTTATAAATGTGATGCATTTGATGCATTCCACAAATAAAGGACCGTATTACAATCAGGTG 120

allele 64 61 TTTATAAATGTGATGCATTTGATGCATTCCACAAATAAAGGACCGTATTACAATCAGGTG 120

allele 48 61 TTTATAAATGTGATGCATTTGATGCATTCCACAAATAAAGGACCGTATTACAATCAGGTG 120

allele 53 61 TTTATAAATGTGATGCATTTGATGCATTCCACAAATAAAGGACCGTATTACAATCAGGTG 120

************************************************************

11K16 121 AATAATTAGGTACCTACTTATCGGAATCGGAACATAGGTACCAAGAATACTTTGAACAGA 180

14B20 121 AATAATTAGGTACCTACTTATCGGAATCGGAACATAGGTACCAAGAATACTTTGAACAGA 180

allele 74 121 AATAATTAGGTACCTACTTATCGGAATCGGAACATAGGTACCAAGAATACTTTGAACAGA 180

allele 58 121 AATAATTAGGTACCTACTTATTGGAATCGGAACATAGGTACCAAGAATACTTTGAACAGA 180

allele 64 121 AATAATTAGGTACCTACTTATCGGAATCGGAACATAGGTACCAAGAATACTTTGAACAGA 180

allele 48 121 AATAATTAGGTACCTACTTATCGGAATCGGAACATAGGTACCAAGAATACTTTGAACAGA 180

allele 53 121 AATAATTAGGTACCTACTTATCGGAATCGGAACATAGGTACCAAGAATACTTTGAACAGA 180

*********************.**************************************

11K16 181 ATATAACTTGCATTGAATATAAATTTACG---TAATTCTTGAACTTGAATCTGATATTTG 237

14B20 181 ATATAACTTGCATTGAATATGAATTTACTCCTCACTACTTGAAGTTGAATCTGATATTTG 240

allele 74 181 ATATAACTTGCATTGAATATAAATTTACG---TAATTCTTGAACTTGAATCTGATATTTG 237

allele 58 181 ATATAACTTGCATTGAATATAAATTTACG---TAATTCTTGAACTTGAATCTGATATTTG 237

allele 64 181 ATATAACTTGCATTGAATATAAATTTACG---TAATTCTTGAACTTGAATCTGATATTTG 237

allele 48 181 ATATAACTTGCATTGAATATAAATTTACG---TAATTCTTGAACTTGAATCTGATATTTG 237

allele 53 181 ATATAACTTGCATTGAATAT----TTACG---TAATTCTTGAACTTGAATCTGATATTTG 233

********************....****. .*.*.******.****************

11K16 238 ATAGCTTACAAGAAGTTGCGATGTTTACCGATACTAACCGATCTAACTAAGTAATTAAAT 297

14B20 241 ATAACTTACAAGAAGTTGCGATGTTTACCGATACTAACCGATCTAACTAAGTAATTAAAT 300

allele 74 238 ATAGCTTACAAGAAGTTGCGATGTTTACCGATACTAACCGATCTAACTAAGTAATTAAAT 297

allele 58 238 ATAGCTTACAAGAAGTTGCGATGTTTACCGATACTAACCGATCTAACTAAGTAATTAAAT 297

allele 64 238 ATAGCTTACAAGAAGTTGCGATGTTTACCGATACTAACCGATCTAACTAAGCAATTAAAT 297

allele 48 238 ATAGCTTACAAGAAGTTGCGATGTTTACCGATACTAACCGATCTAACTAAGTAATTAAAT 297

allele 53 234 ATAGCTTACAAGAAGTTGCGATGTTTACCGATACTAACCGATCTAACTAAGTAATTAAAT 293

***.***********************************************.********

11K16 298 CCATATAGTCAA--TCACTTAATTATGTTTGTTCTTTTTTCCAGATGAACGATACTGTCG 355

14B20 301 CCATATAGTCAA--TCACTTAATTATGTTTGTTCTTTTTTCCAGATGAACGATACTGTCG 358

allele 74 298 CCATATAGTCAA--TCACTTAATTATGTTTGTTCTTTTTTCCAGATGAACGATACTGTCG 355

allele 58 298 CCATATAGTCAA--TCACTTCATTATGTTTGTTCTTTTTTCCAGATGAACGATACTGTCG 355

allele 64 298 CCATATAGTCAA--TCACTTAATTATGTTTGTTCTTTTTTCCAGATGAACGATACTGTCG 355

allele 48 298 CCATATAGTCAAAATCACTTAATTATGTTTGTTCTTTTTTCCAGATGAACGATACTGTCG 357

allele 53 294 CCATATAGTCAA--TCACTTAATTATGTTTGTTCTTTTTTCCAGATGAACGATACTGTCG 351

************ ******.***************************************

11K16 356 ACAAAGTTTGTTCATA-TTATACAAAATTTTCGTTAGGATTAATATTTATTTCGTGCTCA 414

14B20 359 ACAATGTTTGTTCATA-TTATACAAAATTTTCGTTAGGATTAATATTTATTTCGTGCTCA 417

allele 74 356 ACAAAGTTTGTTCATA-TTATACAAAATTTTCGTTAGGATTAATATTTATTTCGTGCTCA 414

allele 58 356 ACAAAGTTTGTTCATA-TTATACAAAATTTTCGTTAGGATTAATATTTATTTCGTGCTCA 414

allele 64 356 ACAAAGTTTGTTCATA-TTATACAAAATTTTCGTTAGGATTAATATTTATTTCGTGCTCA 414

allele 48 358 ACAAAGTTTGTTCATAATTATACAAAATTTTCGTTAAGATTAATATTTATTTCGTGCTCA 417

allele 53 352 ACAAAGTTTGTTCATAATTATACAAAATTTTCGTTAGGATTAATATTTATTTCGTGCTCA 411

****.*********** *******************.***********************

11K16 415 ATGTTTAACGTTGCACCATTCTGTAATAACATTGCAAACGTTTTTATTTTTAAAACTGAA 474

14B20 418 ATGTTTAACGTTGCACCATTCTGTAATAACATTGCAAACGTTTTTATTTTTAAAACTGAA 477

allele 74 415 ATGTTTAACGTTGCACCATTCTGTAATAACATTGCAAACGTTTTTATTTTTAAAACTGAA 474

allele 58 415 ATGTTTAACGTTGCACCATTCTGTAATAACATTGCAAACGTTTTTATTTTTAAAACTGAA 474

allele 64 415 ATGTTTAACGTTGCACCATTCTGTAATAACATTGCAAACGTTTTTATTTTTAAAACTGAA 474

allele 48 418 ATGTTTAACGTTGCACCATTCTGTAATAACATTGCAAACGTTTTTATTTTTAAAACTGAA 477

allele 53 412 ATGTTTAACGTTGCACCATTCTGTAATAACATTGCAAACGTTTTTATTTTTAAAACTGAA 471

************************************************************

11K16 475 AACTACACTTTGGAATTTTCTTTATACTACCAATTTCCCGGAATTGACCCGACCGATTAC 534

14B20 478 AACTACACTTTGGAATTTTCTTTATACTACCAATTTCCCGGAATTGACCCGACCGATTAC 537

allele 74 475 AACTACACTTTGGAATTTTCTTTATACTACCAATTTCCCGGAATTGACCCGACCGATTAC 534

allele 58 475 AACTACACTTTGGAATTTTCTTTATACTACCAATTTCCTGGAATTGACCCGACCGATTAC 534

allele 64 475 AACTACACTTTGGAATTTTCTTTATACTACCAATTTCCTGGAATTGACCCGACCGATTAC 534

allele 48 478 AACTACACCTTGGAATTTTCTTTATACTACCAATTTCCCGGAATTGACCCGACCGATTAC 537

allele 53 472 AACTACACTTTGGAATTTTCTTTATACTACCAATTTCCCGGAATTGACCCGACCGATTAC 531

********.*****************************.*********************

11K16 535 TTCACAACTACTTCGATATACAATTTCTATCTGTCGTACAATTGCGCTATGATGGTGTCT 594

14B20 538 TTCACAACTACTTCGATATACAATTTCTATCTGTCGTACAATTGCGCTATGATGGTGTCT 597

allele 74 535 TTCACAACTACTTCGATATACAATTTCTATCTGTCGTACAATTGCGCTATGATGGTGTCT 594

allele 55 535 TTCACAACTACTTCGATATACAATTTCTATCTGTCGTACAATTGCGCTATGATGGTGTCT 594

allele 58 535 TTCACAACTACTTCGATATACAATTTCTATCTGTCGTACAATTGCGCTATGATGGTGTCT 594

allele 64 535 TTCACAACTACTTCGATATACAATTTCTATCTGTCGTACAATTGCGCTATGATGGTGTCT 594

allele 48 538 TTCACAACTACTTCGATATACAATTTCTATCTGTCGTAC--------------GGTGTCT 583

allele 53 532 TTCACAACTACTTCGATATACAATTTCTATCTGTCGTACAATTGCGCTATGATGGTGTCT 591

***************************************..............*******

11K16 595 GGACTAGATCTAATATTGTTTTTAATAATTTTCCAAATAATCGGGCACGTGTACATCCTG 654

14B20 598 GGACTAGATCTAATATTGTTTTTAATAATTTTCCAAATAATCGGGCACGTGTACATCCTG 657

allele 74 595 GGACTAGATCTAATATTGTTTTTAATAATTTTCCAAATAATCGGGCACGTGTACATCCTG 654

allele 58 595 GGACTAGATCTAATATTGTTTTTAATAATTTTCCAAATAATCGGGCACGTGTACATCCTG 654

allele 64 595 GGACTAGATCTAATATTGTTTTTAATAATTTTCCAAATAATCGGGCACGTGTACATCCTG 654

allele 48 584 GGACTAGATCTAATATTGTTTTTAATAATTTTCCAAATAATCGGGCACGTGTACATCCTG 643

allele 53 592 GGACTAGATCTAATATTGTTTTTAATAATTTTCCAAATAATCGGGCACGTGTACATCCTG 651

************************************************************

11K16 655 AGATACAACCTTGAGAACTTTCCGTGGCCAAAAAATAAAGTGGTTTTCAAACTAGGGGAT 714

14B20 658 AGATACAACCTTGAGAACTTTCCGTGGCCAAAAAATAAAGTGGTTTTCAAACTAGGGGAT 717

allele 74 655 AGATACAACCTTGAGAACTTTCCGTGGCCAAAAAATAAAGTGGTTTCCAAACTAGGGGAT 714

allele 58 655 AGATACAACCTTGAGAACTTTCCGTGGCCAAAAAATAAAGTGGTTTTCAAACTAGGGGAT 714

allele 64 655 AGATACAACCTTGAGAACTTTCCGTGGCCAAAAAATAAAGTGGTTTTCAAACTAGGGGAT 714

allele 48 644 AGATACAACCTTGAGAACTTTCCGTGGCCAAAAAATAAAGTGGTTTTCAAACTAGGGGAT 703

allele 53 652 AGATACAACCTTGAGAACTTTCCGTGGCCAAAAAATAAAGTGGTTTTCAAACTAGGGGAT 711

**********************************************.*************

11K16 715 ATTTTAAAATACAAAATAAACGAGAGCATCACATCCGAAATGTTTGACGCAGAAGAAAAC 774

14B20 718 ATTTTAAAATACAAAATAAACGAGAGCATCACATCCGAAATGTTTGACGCAGAAGAAAAC 777

allele 74 715 ATTTTAAAATACAAAATAAACGAGAGCATCACATCCGAAATGTTTGACGCAGAAGAAAAC 774

allele 58 715 ATTTTAAAATACAAAATAAACGAGAGCATCACATCCGAAATGTTTGACGCAGAAGAAAAC 774

allele 64 715 ATTTTAAAATACAAAATAAACGAGAGCATCACATCCGAAATGTTTGACGCAGAAGAAAAC 774

allele 48 704 ATTTTAAAATACAAAATAAACGAGAGCATCACATCCGAAATGTTTGACGCAGAAGAAAAC 763

allele 53 712 ATTTTAAAATACAAAATAAACGAGAGCATCACATCCGAAATGTTTGACGCAGAAGAAAAC 771

************************************************************

11K16 775 AAA-----------TAAATTGGCAGAATGCATTGAATATCATAAAAAAATAATTGGGTAT 823

14B20 778 AAAGAAGTGCGTTTTAAATTGGCAGAATGCATTGAATATCATAAAAAA-TAATTGGGTAT 836

allele 74 775 AAAGAAGTGCGTTTTAAATTGGCAGAATGCATTGAATATCATAAAAAA-TAATTGGGTAT 833

allele 58 775 AAAGAAGTGCGTTTTAAATTGGCAGAATGCATTGAATATCATAAAAAAATAATTGGGTAT 834

allele 64 775 AAAGAAGTGCGTTTTAAATTGGCAGAATGCATTGAATATCATAAAAAAATAATTGGGTAT 834

allele 48 764 AAAGAAGTGCGTTTTAAATTGGCAGAATGCATTGAATATCATAAAAAAATAATTGGGTAT 823

allele 53 772 AAAGAAGTGCGTTTTAAATTGGCAGAATGCATTGAATATCATAAAAAAATAATTGGGTAT 831

***...........**********************************.***********

11K16 824 GTTGTTGTTATTATATGGTTGTTTGTTCCCACCGAAATAAATTCACAAAACAACGAATGT 883

14B20 837 GTTGTTGTTATTATATGGTTGTTTGTTCCCACCGAAATAAATTCACAAAACAACGAATGT 896

allele 74 834 GTTGTTGTTATTATATGGTTGTTTGTTCCCACCGAAATAAATTCACAAAACAACGAATGT 893

allele 58 835 GTTGTTGTTATTATATGGTTGTTTGTTCCCACCGAAATAAATTCACAAAACAACGAATGT 894

allele 64 835 GTTGTTGTTATTATATGGTTGTTTGTTCCCACCGAAATAAATTCACAAAACAACGAATGT 894

allele 48 824 GTTGTTGTTATTATATGGTTGTTTGTTCCCACCGAAATAAATTCACAAAACAACGAATGT 883

allele 53 832 GTTGTTGTTATTATATGGTTGATTGTTCCCACCGAAATAAATTCACAAAACAACGAATGT 891

*********************.**************************************

11K16 884 CGCCTGAGATAAAGT 898

14B20 897 CGCCTGAGATAAAGT 911

allele 74 894 CGCCTGAGATAAAGT 908

allele 58 895 CGCCTGAGATAAAGT 909

allele 64 895 CGCCTGAGATAAAGT 909

allele 48 884 CGCCTGAGATAAAGT 898

allele 53 892 CGCCTGAGATAAAGT 906

***************
