# Supplementary material for: Sex-Linked Pheromone Receptor Genes of the European Corn Borer, Ostrinia nubilalis, Are in Tandem Arrays
Source: PLoS One. 2011 Apr 22;6(4):e18843. doi: 10.1371/journal.pone.0018843 (PMC3081303; doi:10.1371/journal.pone.0018843)
Supplement: Table S1 — O. nubilalis BACs isolated by PCR-based screening. (DOC) [file pone.0018843.s003.doc]

| Gene | Primer sequences | Product Size (bp) | Positive clones |
| --- | --- | --- | --- |
| *OscaOR1* | CTACTCTCTGCCCTTCAA | 178 | 08K04, 50D02, 65H02 |
|  | CGTCCTCAAATCGTGGTC |  |  |
| *OscaOR2* | GAGACCCTGACTGAACCA | ca. 700 | 07H10 |
|  | ATCCAAAGAAGCACTGAG |  |  |
| *OscaOR3* | GTCTTTGTTGCTTTCGTC | 371 | 08K04, 50D02, 65H02 |
|  | TCATTTCCAGGCTCTCCA |  |  |
| *OscaOR4* | GGATTATGTTGTGCGGATTA | 163 | 01I19, 11K16 |
|  | CAGTTTTCTTTGTTTTTGTA |  |  |
| *OscaOR5/6* | GACCCGAGCGATTACTTCA | 262 | 11K16, 14B20, 41P13, 53I05 |
|  | CTTCTTTGTTTTCTTCTGC |  |  |
| *OscaOR7* | TTCTTACATTTTTCATTCAC | 374 | 11K16, 14B20, 32P24, 41P13, 53I05 |
|  | ATCATTTTGTTCTTCATTTC |  |  |
| *OscaOR8* | CTTGGATTCGTTATGGAC | 1690 | 11K16, 53I05 |
|  | CAGAAAAGCGTAGTATGA |  |  |
| *OnOr6* exons 1–3 | CTCGGCTACATAAAAACCA | ca. 2000 | 01I19, 44E03 |
|  | AACAACAACATTCCTAAGT |  |  |
| *OnOr6* exons 7–8 | TGGGAGATAGATAATGAGACGA | 329 | 01I19, 15L15, 33H07, 44E03 |
|  | ACCGATAATAAGGATACGAACG |  |  |
| 32P24_6 | ATACATAGGGACAGACAGCA | 609 | 32P24, 41P13, 53I05 |
|  | TATTTAGATGAAGGTTAGT |  |  |
