# Supplementary material for: Sex-Linked Pheromone Receptor Genes of the European Corn Borer, Ostrinia nubilalis, Are in Tandem Arrays
Source: PLoS One. 2011 Apr 22;6(4):e18843. doi: 10.1371/journal.pone.0018843 (PMC3081303; doi:10.1371/journal.pone.0018843)
Supplement: Table S2 — BAC probes used for FISH analysis. (DOC) [file pone.0018843.s004.doc]

| ***O. nubilalis* genes and ESTs^1^** | **BACs used** | **FISH probes (Fig. 3)** | | **Accession No. of *B. mori* ortholog** | **Chr.** | **Location in Kaikobase ^4^** | |
| --- | --- | --- | --- | --- | --- | --- | --- |
|  |  | **Dye^2^** | **Pseudocolor^3^** |  |  |  |  |
| kettin | 05P02 | O^*^ | Oy | AB079865 | Z | 6524661 - 6525956 | |
| FTZ-F1 | 22G03 | C5 | M | D10953 | Z | 11667857-11753363 | |
| *OnOr6* | 44E03 | G^4^ | G |  | | | |
| *OnubOR5* | 14B20 | O | Y |  |  |  |  |
| *OnubOR7* |  |  |  |  |  |  |  |
| *OnubOR7* | 32P24 | G^**^ | R |  |  |  |  |
| lactate dehydrogenase | 23A08 | G | Lb | EU000385 | Z | | 17338625-17350605 |
| *OnubOR2* | 07H10 | C5 | M | AJ555487 | 16 | | 4359199 - 4348221 |
| EL929838 | 24F10 | G | G |  | 16 | | 12311195-12304348 |
| EL929540 |  |  |  | DQ443264 | 16 | | 12491599-12490079 |
| *OnubOR1* | 50D02 | C5 | M |  | | | |
| *OnubOR3* |  |  |  |  |  |  |  |
| FS438619 | 25M12 | G | G | AB196700 | 23 | | 11155444-11160627 |
| FS438672 | 26B09 | R | R |  | 23 | | 12123813-12113865 |
| EL929158 | 29L07 | O | O | DQ515927 | 23 | | 15066258-15082587 |

1: Accession numbers are listed to identify ESTs.

2: BAC probes labeled with Cy5-dUTP (C5), Green-dUTP (G), Orange-dUTP (O) and Red-dUTP R) respectively

3 The signals from BAC probes pseudocolored with G: green, Lb: light blue, M: magenta, Oy: orange yellow, R: red and Y: yellow

4: Inferred from <http://sgp.dna.affrc.go.jp/KAIKObase/> * 1st round of reprobed FISH ** 2nd round of reprobed FISH
