# Supplementary material for: Sex-Linked Pheromone Receptor Genes of the European Corn Borer, Ostrinia nubilalis, Are in Tandem Arrays
Source: PLoS One. 2011 Apr 22;6(4):e18843. doi: 10.1371/journal.pone.0018843 (PMC3081303; doi:10.1371/journal.pone.0018843)
Supplement: Table S4 — Chromosomal distribution of OR genes in B. mori. (DOC) [file pone.0018843.s006.doc]

Table S4 Chromosomal distribution of OR genes in *B. mori*.

| Name | Accession No. | Chr. | Start position | End position |
| --- | --- | --- | --- | --- |
| Or1 | AB059431 | Z | 12375121 | 12385059 |
| Or4 | AB186506 | Z | 16437671 | 16432659 |
| Or9 | BK005919 | Z | 16447935 | 16441110 |
| Or3 | AB186505 | Z | 22372604 | 22378542 |
| Or35 | BK005941 | 3 | 317084 | 319882 |
| Or38 | BK005944 | 3 | 1686017 | 1691763 |
| Or39 | BK005945 | 3 | 1689705 | 1687382 |
| Or13 | BK005923 | 5 | 7701551 | 7699903 |
| Or12 | BK005922 | 5 | 7717149 | 7713998 |
| Or61 | AB472138 | 5 | 7729196 | 7725663 |
| Or15 | BK005924 | 5 | 7735927 | 7732901 |
| Or59 | AB472136 | 5 | 20907382 | 20913112 |
| Or26 | AB472106 | 5 | 20915096 | 20917793 |
| Or7 | AJ874109 | 6 | 17235416 | 17248288 |
| Or5 | AB186507 | 6 | 17263698 | 17272416 |
| Or53 | AB472130 | 7 | 11343890 | 11347233 |
| Or27 | BK005934 | 7 | 11355177 | 11355955 |
| Or63 | AB472140 | 8 | 1328159 | 1346351 |
| Or45 | BK005951 | 8 | 1363363 | 1348275 |
| Or58 | AB472135 | 8 | 1390172 | 1401633 |
| Or30 | BK005936 | 9 | 9780335 | 9782848 |
| Or60 | AB472137 | 9 | 10506263 | 10511987 |
| Or66 | AB472143 | 9 | 13308017 | 13298224 |
| Or65 | AB472142 | 9 | 13360683 | 13357069 |
| Or64 | AB472141 | 9 | 13661238 | 13682188 |
| Or19 | BK005927 | 9 | 17905328 | 17911480 |
| Or20 | BK005928 | 9 | 17912802 | 17917803 |
| Or28 | AB472108 | 10 | 4770729 | 4772192 |
| Or54 | AB472131 | 10 | 4874572 | 4870098 |
| Or55 | AB472132 | 10 | 4883463 | 4879783 |
| Or6 | AB186508 | 12 | 4417361 | 4426517 |
| Or32 | BK005938 | 12 | 12159773 | 12158929 |
| Or25 | BK005933 | 13 | 10876196 | 10882805 |
| Or48 | BK005954 | 16 | 2836177 | 2847547 |
| Or57 | AB472134 | 16 | 2873832 | 2854982 |
| Or46 | FJ170765 | 16 | 2904270 | 2895599 |
| Or2 | AB100454 | 16 | 4359029 | 4348257 |
| Or52 | AB472129 | 16 | 13755509 | 13768695 |
| Or10 | BK005920 | 19 | 362800 | 355634 |
| Or49 | AB472126 | 19 | 7057408 | 7056809 |
| Or42 | BK005948 | 21 | 7352455 | 7358095 |
| Or67 | AB472144 | 21 | 8539418 | 8546352 |
| Or8 | AB472091 | 21 | 10612119 | 10620678 |
| Or21 | BK005929 | 21 | 10658516 | 10667672 |
| Or22 | BK005930 | 21 | 10671228 | 10675402 |
| Or44 | BK005950 | 21 | 10954898 | 10956557 |
| Or17 | AB472098 | 21 | 16543928 | 16538765 |
| Or50 | AB472127 | 22 | 16212797 | 16169029 |
| Or51 | AB472128 | 22 | 17980882 | 17977541 |
| Or36 | BK005942 | 22 | 19939287 | 19936893 |
| Or29 | BK005935 | 22 | 23059644 | 23058871 |
| Or14 | DQ991151 | 23 | 18643653 | 18646103 |
| Or41 | BK005947 | 25 | 11124161 | 11118163 |
| Or40 | BK005946 | 25 | 11130205 | 11127533 |
| Or16 | AB472097 | 25 | 11152232 | 11147067 |
| Or37 | BK005943 | 25 | 16663235 | 16665974 |
| Or18 | AB472099 | 26 | 8491113 | 8494755 |
| Or68 | AB472145 | 27 | 3292429 | 3291919 |
| Or34 | AB472113 | 27 | 3321945 | 3321181 |
| Or33 | AB472112 | 27 | 3321945 | 3317100 |
| Or49* | EU779802 | 28 | 3406516 | 3411066 |
| Or11 | BK005921 | 28 | 6804757 | 6799853 |
| Or24 | BK005932 | 28 | 6813999 | 6812345 |
| Or23 | BK005931 | 28 | 6834845 | 6830085 |
| Or56 | AB472133 | 28 | 9250334 | 9236318 |
| Or62 | AB472139 | unmapped | | |

*Gene names are according to Tanaka et al. 2009, with the exception of independently named Or49(EU779802).
